# Limited Effect of 60-Days Strict Head Down Tilt Bed Rest on Vascular Aging

**DOI:** 10.3389/fphys.2021.685473

**Published:** 2021-05-28

**Authors:** Stefan Möstl, Stefan Orter, Fabian Hoffmann, Martin Bachler, Bernhard Hametner, Siegfried Wassertheurer, Jérémy Rabineau, Edwin Mulder, Bernd Johannes, Jens Jordan, Jens Tank

**Affiliations:** ^1^German Aerospace Center (DLR), Institute of Aerospace Medicine, Cologne, Germany; ^2^Center for Health and Bioresources, AIT Austrian Institute of Technology, Vienna, Austria; ^3^Department of Cardiology, University Hospital Cologne, Cologne, Germany; ^4^Laboratory of Physics and Physiology, University of Brussels, Brussels, Belgium; ^5^Chair of Aerospace Medicine, University Hospital Cologne, Cologne, Germany

**Keywords:** pre-ejection period, pulse wave arrival time, pulse wave velocity, artificial gravity, AGBRESA, arterial stiffness, aortic distensibility, isovolumetric contraction time

## Abstract

**Background:**

Cardiovascular risk may be increased in astronauts after long term space flights based on biomarkers indicating premature vascular aging. We tested the hypothesis that 60 days of strict 6° head down tilt bed rest (HDTBR), an established space analog, promotes vascular stiffening and that artificial gravity training ameliorates the response.

**Methods:**

We studied 24 healthy participants (8 women, 24–55 years, BMI = 24.3 ± 2.1 kg/m^2^) before and at the end of 60 days HDTBR. 16 subjects were assigned to daily artificial gravity. We applied echocardiography to measure stroke volume and isovolumetric contraction time (ICT), calculated aortic compliance (stroke volume/aortic pulse pressure), and assessed aortic distensibility by MRI. Furthermore, we measured brachial-femoral pulse wave velocity (_bf_PWV) and pulse wave arrival times (PAT) in different vascular beds by blood pressure cuffs and photoplethysmography. We corrected PAT for ICT (cPAT).

**Results:**

In the pooled sample, diastolic blood pressure (+8 ± 7 mmHg, *p* < 0.001), heart rate (+7 ± 9 bpm, *p* = 0.002) and ICT (+8 ± 13 ms, *p* = 0.036) increased during HDTBR. Stroke volume decreased by 14 ± 15 ml (*p* = 0.001). _bf_PWV, aortic compliance, aortic distensibility and all cPAT remained unchanged. Aortic area tended to increase (*p* = 0.05). None of the parameters showed significant interaction between HDTBR and artificial gravity training.

**Conclusion:**

60 days HDTBR, while producing cardiovascular deconditioning and cephalad fluid shifts akin to weightlessness, did not worsen vascular stiffness. Artificial gravity training did not modulate the response.

## Introduction

Vascular biomarker studies suggest that the harsh environmental conditions in space including microgravity, galactic cosmic radiation, and perturbed circadian rhythms may promote vascular aging. Premature vascular aging could pose risks for astronauts’ performance and cardiovascular health during missions to the Moon and from there to Mars. Indeed, decreased carotid distensibility (Hughson et al., 2016; Arbeille et al., 2017), increased carotid and femoral intima media thickness (Arbeille et al., 2016), and decreased pulse wave arrival times (PAT) at the finger (Baevsky et al., 2007; Hughson et al., 2016) have been reported following long-term space missions. PAT relates to pulse wave velocity (PWV), an established cardiovascular risk marker (Williams et al., 2018), provided that isovolumetric contraction time (ICT) and vessel length do not change.

Beside aging, physical inactivity during bed rest rapidly evokes blood vessel remodeling (Thijssen et al., 2011). Sixty days head down tilt bed rest (HDTBR), which additionally models cardiovascular deconditioning and cephalad fluid shifts in space, also increased carotid and femoral intima media thickness (van Duijnhoven et al., 2010) and aortic PWV (Fayol et al., 2019). In another 35 days HDTBR study, the same vascular stiffness markers did not change (Palombo et al., 2015). Physical exercise reduced intima media thickening during HDTBR (van Duijnhoven et al., 2010). Artificial gravity through daily short-arm centrifugation, which partially attenuated cardiovascular deconditioning during 21 days HDTBR (Stenger et al., 2012), also holds promise in this regard.

Therefore, we hypothesized that 60 days HDTBR would increase aortic PWV and that artificial gravity would attenuate the response. Moreover, we recorded PAT at different sites to test for regional differences in vascular adaptation. Finally, unlike previous studies, we assessed distensibility and compliance of the aortic arch with magnetic resonance imaging and echocardiography, respectively.

## Materials and Methods

### Participants

After providing written informed consent, 8 women and 16 men aged between 24 and 55 years (33.4 ± 9.3 years, 24.3 ± 2.1 kg/m^2^) participated in the Artificial Gravity Bed Rest Study (AGBRESA). AGBRESA was a joint project between the National Aeronautics and Space Administration (NASA), the European Space Agency (ESA), and the German Aerospace Center (DLR). All participants had no history of cardiovascular disease, took no medication, and were non-smokers for at least 6 months prior to enrollment. The study was conducted in accordance with the declaration of Helsinki and was registered at the German Clinical Trials Register under number DRKS00015677. The protocol was approved by the ethics commissions of the Medical Association North Rhine (number 2018143) and NASA (Johnson Space Center, Houston, United States).

### Study Design

**FIGURE 1:**
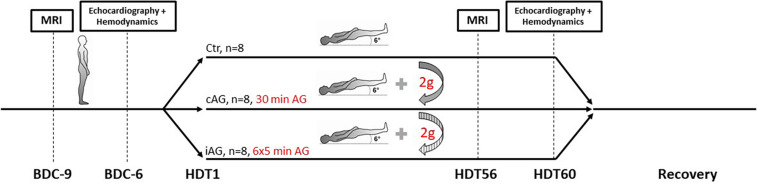
Illustration of the study design. During 14-day baseline data collection (BDC), subjects were ambulatory but supine for measurements. Shortly before the onset of the 60-day head down tilt period (HDT), subjects were assigned to three different intervention groups named control (Ctr, no artificial gravity), continuous artificial gravity (cAG), and intermittent artificial gravity (iAG). We obtained magnetic resonance imaging (MRI) measurements 9 days before (BDC-9), and on the 56th day of HDT (HDT56). We conducted echocardiography and assessed hemodynamics (blood pressure, pulse wave velocity, etc.) on BDC-6 and on HDT60.

The overreaching goal of AGBRESA was to assess the efficacy of artificial gravity as countermeasure for physiological adaptations evoked by 60 days strict HDTBR. Strict HDTBR means that no pillows were allowed except for a thin cushion when participants lay on the side, one shoulder always had to touch the mattress, and all daily activities including personal hygiene were done in the −6° position. Including the pre and post bed rest phases, the participants spent 87 days at the Envihab research facility in Cologne, Germany. All participants ingested standardized isocaloric diets with controlled fluid intake and were subjected to regulated bed times. Caffeine or alcohol containing beverages were not allowed. Participants did not exercise at least 24 h before we obtained our recordings. After the 14-day base line data collection (BDC), participants were distributed to three groups subjected to no artificial gravity (control, Ctr), continuous artificial gravity (cAG), or intermittent artificial gravity (iAG) (Figure 1). The bed rest period was followed by 14 days of recovery.

### Artificial Gravity Countermeasure

Artificial gravity was generated by a short arm centrifuge with participants in the supine position and the head toward the center of rotation. Distance to the center of rotation and angular velocity were adjusted to body height to reach 1 g at the center of body mass, and approximately 2 g at the feet. Daily artificial gravity was applied continuously for 30 min (cAG) or intermittently for 6 × 5 min (iAG) with 3-min breaks in between. Rotation direction was changed daily. A more detailed description of the artificial gravity countermeasure can be found elsewhere (Kramer et al., 2020).

### Instrumentation and Experimental Protocol

**FIGURE 2:**
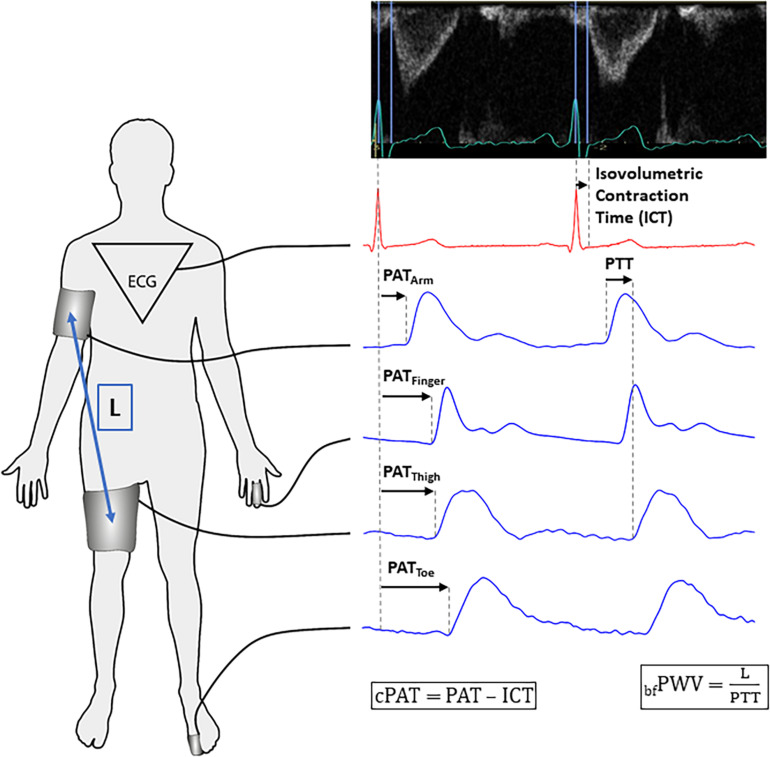
Data acquisition for vascular measurements. **Left panel:** Recording sites and length (L) determination for brachial-femoral pulse wave velocity (_bf_PWV) assessment. **Right panel – top:** ECG and Doppler flow curves in the left ventricular outflow tract. We measured isovolumetric contraction time (ICT) from the R-peak to aortic valve opening indicated by flow onset. **Right panel – bottom:** Pulse wave arrival time (PAT) is defined as the time from the ECG R-Peak to pulse wave arrival at a certain landmark. By dividing L by pulse transit time (PTT) between arm and thigh cuffs, we obtained _bf_PWV. We obtained measurements from every heartbeat recorded during a 60-s period. For the sake of clarity, PAT and PTT are shown for two consecutive heart beats. We calculated corrected PAT (cPAT) by subtracting ICT from PAT.

We obtained recordings 6 days before (BDC-6, PRE) and on the last day of HDTBR (HDT60, HDT) following a complete clinical echocardiographic examination (Vivid-IQ with M5SC-RS sector-probe, GE Healthcare, Buckinghamshire, Great Britain) according to current guidelines (Galderisi et al., 2017). We assessed ICT as the interval between ventricular contraction onset, indicated by the electrocardiogram R-peak, and aortic valve opening, as indicated by flow onset in the left ventricular outflow tract (Figure 2). We calculated stroke volume by multiplying velocity time integral with left ventricular outflow tract area. For the PRE-sessions, participants were in the horizontal supine position. At HDT60, the echocardiography table was tilted to **−**6° in order to maintain strict HDTBR.

After the echocardiographic examination, we instrumented participants as shown in Figure 2. We placed a finger blood pressure cuff (Finometer MIDI, Finapres Medical Systems, Enschede, Netherlands) around the left ring or middle finger and recorded its raw analog signals at 2000 Hz (BIOPAC MP150, Biopac Systems Inc., Goleta, United States). We also placed blood pressure cuffs at the right upper arm and right thigh. During the PRE-sessions, we measured the distance between these cuffs to exactly reposition them at HDT60 (Figure 2). These cuffs were connected to a research device for non-invasive hemodynamic analysis (CardioCube, AIT Austrian Institute of Technology, Vienna, Austria). Beside the option to simultaneously operate two blood pressure cuffs, the device features a 3-lead-electrocardiogram module and two analog input channels recording signals at 250 Hz. The first channel recorded the pulse curve obtained with a photoplethysmography sensor (Blood Volume Pulse, PLUX wireless biosignals, Arruda dos Vinhos, Portugal) attached to the left long toe. The second channel recorded a trigger signal synchronizing CardioCube with Biopac. After instrumentation, we obtained three oscillometric blood pressure measurements using the upper arm cuff to record heart rate and brachial systolic and diastolic blood pressure (SBP and DBP). The CardioCube also calculated aortic systolic and diastolic blood pressure using the ARCSolver algorithm (Weber et al., 2011). Then, the cuffs were inflated to the previously determined DBP to record pulse curves and electrocardiogram for 1 min (Figure 2).

### Signal and Data Analysis

We determined PAT as the time between electrocardiogram R-Peak and pulse wave arrival at the upper arm (PAT_Arm_), finger (PAT_Finger_), thigh (PAT_Thigh_), and toe (PAT_Toe_) (Figure 2). For precise determination of pulse wave upstroke onset, we applied the so-called “diastole-patching method” to find the pairwise maximum cross correlation (implemented with MATLAB 2018, The MathWorks Inc., Natick, United States) (Bachler et al., 2013).

Because PAT could be confounded by ICT, we subtracted ICT from PAT values to obtain corrected measurements cPAT_Arm_, cPAT_Finger_, cPAT_Thigh_, and cPAT_Toe_. As aortic PWV surrogate, we determined brachial-femoral PWV (_bf_PWV) by dividing the distance between arm and thigh cuffs by pulse transit time between these cuffs (Baier et al., 2018). Cardiac output was calculated by multiplying stroke volume with heart rate. Brachial pulse pressure was calculated by subtracting DBP from SBP. Aortic compliance (AOC) was obtained by dividing stroke volume by aortic pulse pressure (aortic SBP – aortic DBP).

### Magnetic Resonance Imaging

We obtained aortic distensibility (AOD) using magnetic resonance imaging at BDC-9 (PRE) and HDT56 (HDT). To measure aortic cross-sectional area changes during one heart cycle, we applied two-dimensional steady-state-free-precession (SSFP) cine imaging (3 Tesla mMR Biograph PET/MRI scanner, Siemens Healthineers, Erlangen, Germany). We also measured brachial blood pressure at the right arm (Expression MR400, Philips Healthcare, Eindhoven, Netherlands) to obtain pulse pressure during the magnetic resonance imaging session. Then, we calculated AOD as relation between cross-sectional area changes of the ascending aorta and brachial pulse pressure (Voges et al., 2012). We also report the end diastolic minimum of the aortic area (AOA_min_) since aortic dilation may influence stiffness (Guala et al., 2019).

### Statistical Analysis

Linear mixed effects (LME) model (IBM SPSS, Version 21) was used for statistical analysis. Given the strong effect of age on vascular stiffness, we conducted an exploratory analysis by stratifying participants in age tertiles, which resulted in three different age groups: age group 1, 24–27 years, *n* = 9; age group 2, 29–36 years, *n* = 8; and age group 3, 37–55, *n* = 7. Bed rest (PRE vs. HDT) and its interaction with age group, sex, and intervention (Ctr, cAG, and iAG) were defined as fixed effect whereas the subjects were defined as random effects. In case of significant interaction, we identified affected groups using Bonferroni *post-hoc* correction for the multiple pairwise comparisons of PRE vs. HDT. A *p*-value < 0.05 indicated statistical significance. Results are reported as measured mean values ± standard deviation. We used Kolmogorov–Smirnov test for checking the distribution of residuals calculated by LME analysis.

## Results

### Hemodynamic and Vascular Response

Because baseline characteristic and cardiovascular responses did not differ between interventions, we conducted a pooled analysis (Table 1). All results sorted by intervention are provided in Table 2. Over all subjects, SBP remained unchanged after 60 days HDTBR (*p* = 0.652), whereas DBP increased from 70 ± 7 mmHg to 78 ± 7 (*p* < 0.001), such that pulse pressure decreased by 9 ± 9 mmHg (*p* < 0.001). On the fourth day of recovery, DBP had returned to baseline values at 70 ± 6 mmHg. To verify these findings, we also analyzed safety blood pressure measurements at PRE and HDT59 obtained through an oscillometric device (IntelliVue X2, Philips Healthcare, Eindhoven, The Netherlands). These measurements were obtained in duplicate immediately after awakening. At HDT59, DBP was increased by 7 ± 7 mmHg (*p* < 0.001). Aortic blood pressure followed brachial blood pressure with an unchanged aortic SBP (*p* = 0.935) and an increased aortic DBP (9 ± 7 mmHg, *p* < 0.001). A 14 ± 15 ml (*p* = 0.001) stroke volume reduction was partly compensated by a heart rate increase (7 ± 9 bpm, *p* = 0.002) such that cardiac output remained stable (5.9 ± 0.9 vs. 5.6 ± 1.1 l/min, *p* = 0.265). AOC remained unchanged following HDTBR (*p* = 0.094). While AOA_min_ tended to increase at the end of HDTBR (Table 1), we observed no changes in AOD (*p* = 0.364).

**TABLE 1:** All recorded parameters sorted by recordings before (PRE) and at the end of head down tilt bed rest (HDT).

Parameter	Pooled analysis (*n* = 24)			*p*-value
	PRE	Δ (HDT-PRE)	HDT	
Aortic SBP (mmHg)	108 ± 12	0 ± 11	108 ± 8	0.935
Aortic DBP (mmHg)	72 ± 7	+ 9 ± 7	81 ± 7	**<0.001**
SBP (mmHg)	125 ± 11	−1 ± 10	124 ± 9	0.652
DBP (mmHg)	70 ± 7	+ 8 ± 7	78 ± 7	**<0.001**
Pulse pressure (mmHg)	55 ± 13	−9 ± 9	46 ± 9	**<0.001**
Heart rate (bpm)	62 ± 9	+ 7 ± 9	69 ± 11	**0.002**
Stroke volume (ml)	97 ± 20	−14 ± 15	83 ± 15	**0.001**
Cardiac output (l/min)	5.9 ± 0.9	−0.3 ± 0.8	5.6 ± 1.1	0.265
PAT_Arm_ (ms)	154 ± 15	+ 9 ± 12	163 ± 17	**0.002**
PAT_Finger_ (ms)	205 ± 17	−1 ± 20	204 ± 18	0.898
PAT_Thigh_ (ms)	209 ± 19	+ 8 ± 15	217 ± 19	**0.022**
PAT_Toe_ (ms)	353 ± 22	−3 ± 27	350 ± 24	0.957
ICT (ms)	49 ± 8	+ 8 ± 13	57 ± 11	**0.036**
cPAT_Arm_ (ms)	105 ± 14	+ 2 ± 15	107 ± 15	0.354
cPAT_Finger_ (ms)	156 ± 17	−8 ± 19	148 ± 13	0.058
cPAT_Thigh_ (ms)	160 ± 19	0 ± 16	160 ± 17	0.561
cPAT_Toe_ (ms)	304 ± 18	+ 11 ± 24	293 ± 22	0.188
_bf_PWV (m/s)	9.0 ± 1.2	+ 0.2 ± 1.0	9.2 ± 1.3	0.864
AOC (ml/mmHg)	2.7 ± 0.6	+ 0.4 ± 0.8	3.1 ± 0.6	0.094
AOD (10^–3^ mm Hg^–1^)	4.8 ± 2.0	−0.5 ± 2.4	4.3 ± 2.2	0.364
AOA_min_ (mm^2^)	481 ± 88	+ 24 ± 53	505 ± 102	0.050

*Aortic SBP and aortic DBP, aortic systolic and diastolic blood pressure; SBP and DBP, brachial systolic and diastolic blood pressure; PAT, pulse wave arrival time; ICT, isovolumetric contraction time; cPAT, PAT corrected for ICT; _bf_PWV, brachial-femoral pulse wave velocity; AOC, aortic compliance; AOD, aortic distensibility; AOA_min_, minimum aortic area. Results are represented as mean values ± standard deviation from 24 subjects and statistical significance (p < 0.05) is indicated by p-values in bold.*

**TABLE 2:** All parameters recorded before (PRE) and at the end of head down tilt bed rest (HDT), sorted by intervention: control (Ctr), continuous artificial gravity (cAG) and intermittent artificial gravity (iAG).

Parameter	Ctr (*n* = 8, 2 women)		cAG (*n* = 8, 3 women)		iAG (*n* = 8, 3 women)		*p*-values	
	PRE	HDT	PRE	HDT	PRE	HDT	Main effect	Interaction
Aortic SBP (mmHg)	112 ± 10	113 ± 8	109 ± 17	108 ± 7	104 ± 7	105 ± 5	0.116	0.463
Aortic DBP (mmHg)	73 ± 9	82 ± 10	72 ± 7	81 ± 5	70 ± 7	79 ± 3	0.680	0.803
SBP (mmHg)	125 ± 8	129 ± 8	127 ± 15	124 ± 11	123 ± 10	120 ± 6	0.542	0.050
DBP (mmHg)	71 ± 8	80 ± 10	70 ± 6	79 ± 5	68 ± 8	76 ± 3	0.532	0.921
Pulse pressure (mmHg)	54 ± 8	49 ± 10	57 ± 15	45 ± 9	55 ± 15	44 ± 7	0.773	0.112
Heart rate (bpm)	62 ± 8	70 ± 10	63 ± 12	71 ± 15	62 ± 7	65 ± 10	0.710	0.162
Stroke volume (ml)	99 ± 27	84 ± 12	94 ± 21	78 ± 11	99 ± 14	87 ± 20	0.683	0.712
Cardiac output (l/min)	6.0 ± 1.3	5.9 ± 0.9	5.7 ± 0.7	5.5 ± 1.0	6.0 ± 0.8	5.6 ± 1.5	0.859	0.706
PAT_Arm_ (ms)	160 ± 14	173 ± 19	153 ± 14	160 ± 14	149 ± 16	156 ± 15	0.125	0.993
PAT_Finger_ (ms)	209 ± 15	214 ± 17	208 ± 22	198 ± 19	199 ± 15	201 ± 13	0.390	0.582
PAT_Thigh_ (ms)	215 ± 14	222 ± 17	205 ± 19	208 ± 18	208 ± 24	221 ± 22	0.188	0.376
PAT_Toe_ (ms)	349 ± 17	354 ± 29	360 ± 26	347 ± 26	349 ± 23	350 ± 21	0.716	0.443
ICT (ms)	50 ± 10	63 ± 12	50 ± 7	54 ± 11	47 ± 6	54 ± 9	0.307	0.445
cPAT_Arm_ (ms)	110 ± 14	110 ± 18	103 ± 15	106 ± 14	101 ± 14	103 ± 12	0.218	0.410
cPAT_Finger_ (ms)	159 ± 16	151 ± 11	158 ± 22	145 ± 18	151 ± 10	147 ± 9	0.811	0.447
cPAT_Thigh_ (ms)	165 ± 16	160 ± 11	155 ± 20	154 ± 16	161 ± 22	167 ± 22	0.343	0.063
cPAT_Toe_ (ms)	300 ± 12	291 ± 25	310 ± 21	290 ± 23	301 ± 21	297 ± 22	0.555	0.405
_bf_PWV (m/s)	9.0 ± 1.1	9.2 ± 1.5	9.1 ± 1.0	9.5 ± 0.8	9.0 ± 1.5	9.0 ± 1.6	0.664	0.875
AOC (ml/mmHg)	2.6 ± 0.6	2.8 ± 0.2	2.8 ± 0.7	3.0 ± 0.5	2.9 ± 0.3	3.4 ± 0.7	0.176	0.386
AOD (10^–3^ mm Hg^–1^)	5.2 ± 2.4	4.4 ± 2.9	4.7 ± 2.1	4.5 ± 1.9	4.6 ± 1.7	4.1 ± 1.9	0.465	0.865
AOA_min_ (mm^2^)	496 ± 70	504 ± 107	456 ± 79	487 ± 52	492 ± 116	522 ± 138	0.630	0.317

*Aortic SBP and aortic DBP, aortic systolic and diastolic blood pressure; SBP and DBP, brachial systolic and diastolic blood pressure; PAT, pulse wave arrival time; ICT, isovolumetric contraction time; cPAT, PAT corrected for ICT; _bf_PWV, brachial-femoral pulse wave velocity; AOC, aortic compliance; AOD, aortic distensibility; AOA_min_, minimum aortic area. Each parameter is represented as mean value ± standard deviation together with p-values for main effects (intervention) and interaction (intervention vs. bed rest).*

### Pulse Wave Velocity and Arrival Times

**FIGURE 3:**
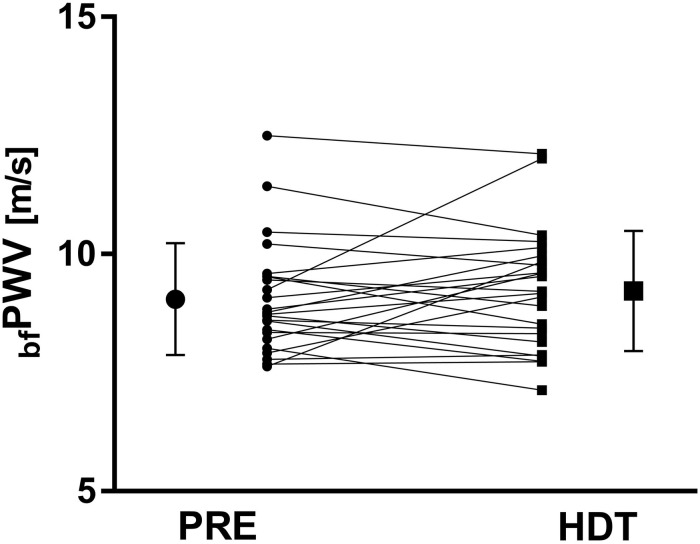
Line graph for individual brachial-femoral pulse wave velocities (_bf_PWV) 6 days before (PRE, dot symbol) and after 60 days HDTBR (HDT, square symbol). Scaled-up single symbols to the left and right represent corresponding mean values and error bars indicate the first standard deviation. _bf_PWV did not change with 60 days HDTBR (9.0 ± 1.2 vs. 9.2 ± 1.3 m/s, *p* = 0.864).

**FIGURE 4:**
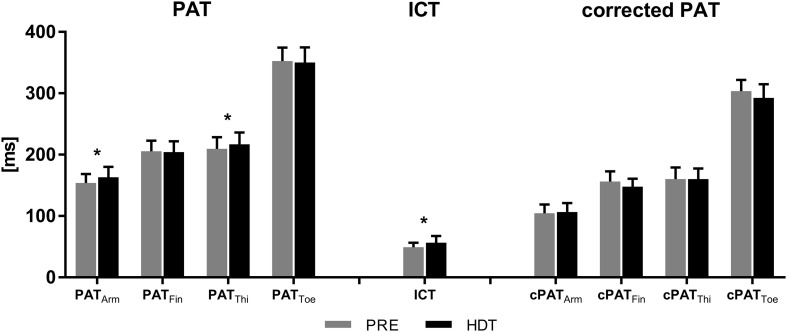
Pulse wave arrival times (PAT) increase with greater topological distance from the heart to arm, finger, thigh, and toe, all indicated by subscripted acronyms. PAT_Arm_ and PAT_Thigh_ significantly increased after 60 days HDTBR (black bars) compared to baseline recordings 6 days before HDTBR (gray bars). Isovolumetric contraction time (ICT) also increased with HDTBR. Corrected PAT did not change with HDTBR. Results are represented as mean values from 24 subjects with error bars indicating the first standard deviation; * < 0.05.

Averaged over all subjects, _bf_PWV remained unchanged between PRE and HDT (*p* = 0.864, Figure 3). In contrast, the uncorrected arrival times at brachial and femoral artery, which include the ICT, increased by 9 ± 13 ms (PAT_Arm_, *p* = 0.002) and 8 ± 15 ms (PAT_Thigh_, *p* = 0.022), respectively. PAT_Finger_ and PAT_Toe_ remained unchanged (*p* = 0.898 and 0.957). However, ICT increased by 8 ± 13 ms (*p* = 0.036) after 60 days in HDTBR and the corrected PAT values (see Figure 4) for the femoral and brachial artery (cPAT_Thigh_ and cPAT_Arm_) were unaffected by HDTBR. In contrast, cPAT_Finger_ and cPAT_Toe_ tended to decrease by 8 ± 5 and 11 ± 13 ms, without reaching significance after correction for the increased ICT (see Table 1).

### Potential Age and Sex Influences

**FIGURE 5:**
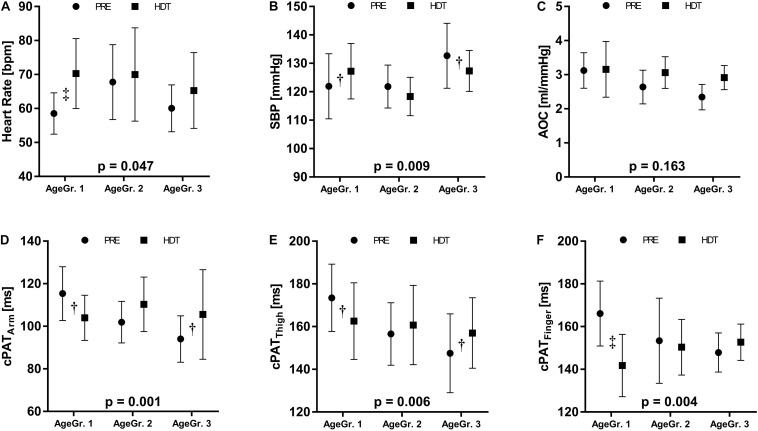
Exploratory analysis relating the HDTBR response to age. We stratified participants in three age groups (AgeGr.): AgeGr. 1 = 24–27 years; AgeGr. 2 = 29–36 years, and AgeGr. 3 = 37–55 years. **(A)** heart rate, **(B)** systolic blood pressure (SBP), **(C)** aortic compliance (AOC), and corrected pulse wave arrival times to the **(D)** arm (cPAT_Arm_), **(E)** thigh (cPAT_Thigh_), and **(F)** finger (cPAT_Finger_). The *p*-values indicate the interaction between AgeGr. and bed rest. Results are represented as mean values ± standard deviation from base line recordings (PRE, dot symbol) and HDT (square symbol). Significant difference between these time points is indicated by ^†^(*p* < 0.05) and ‡(*p* < 0.001).

In general, AOA_min_ and _bf_PWV increased from the youngest to the oldest tertile (*p* = 0.013 and *p* = 0.027). For some of the measurements, we observed a significant interaction between age and HDTBR. These results are listed in Table 3 and an exemplary selection is displayed in Figure 5. We did not observe a qualitative difference in the response to HDTBR between women and men (Table 4).

**TABLE 3:** All parameters recorded before (PRE) and at the end of head down tilt bed rest (HDT).

	AgeGr. 1 (*n* = 9, 2 Women)		AgeGr. 2 (*n* = 8, 4 Women)		AgeGr. 3 (*n* = 7, 2 Women)		*p*-values	
	PRE	HDT	PRE	HDT	PRE	HDT	Main effect	Interaction
Aortic SBP (mmHg)	104 ± 7	110 ± 9	104 ± 8	103 ± 4	119 ± 14	113 ± 5	**0.004**	0.070
Aortic DBP (mmHg)	69 ± 6	82 ± 8	72 ± 5	79 ± 4	75 ± 10	82 ± 7	0.538	0.096
SBP (mmHg)	122 ± 12^†^	127 ± 10	122 ± 8	118 ± 7	133 ± 11^†^	127 ± 7	0.071	**0.009**
DBP (mmHg)	67 ± 6	80 ± 9	70 ± 4	76 ± 4	73 ± 11	79 ± 8	0.569	0.144
Pulse pressure (mmHg)	55 ± 13	48 ± 9	52 ± 10	42 ± 5	60 ± 15	48 ± 11	0.527	0.217
Heart rate (bpm)	59 ± 6^‡^	70 ± 10	68 ± 11	70 ± 14	60 ± 7	65 ± 11	0.665	**0.047**
Stroke volume (ml)	108 ± 21	84 ± 17	83 ± 19	74 ± 11	100 ± 11	91 ± 13	0.129	0.093
Cardiac output (l/min)	6.3 ± 1.2	5.9 ± 1.4	5.5 ± 0.7	5.2 ± 1.0	5.9 ± 0.3	5.9 ± 0.7	0.572	0.830
PAT_Arm_ (ms)	161 ± 15	163 ± 18	154 ± 10^†^	162 ± 11	144 ± 15^†^	164 ± 23	0.441	**0.014**
PAT_Finger_ (ms)	212 ± 16	201 ± 20	205 ± 21	202 ± 15	198 ± 14	211 ± 18	0.978	0.095
PAT_Thigh_ (ms)	220 ± 18	222 ± 21	208 ± 15	212 ± 16	198 ± 19	216 ± 22	0.158	0.086
PAT_Toe_ (ms)	351 ± 21	342 ± 22	357 ± 18	359 ± 17	349 ± 29	353 ± 32	0.167	0.567
ICT (ms)	46 ± 7	59 ± 10	52 ± 8	52 ± 13	50 ± 7	59 ± 9	0.849	0.187
cPAT_Arm_ (ms)	115 ± 13^†^	104 ± 11	102 ± 10	110 ± 13	94 ± 11^†^	106 ± 21	0.203	**0.001**
cPAT_Finger_ (ms)	166 ± 15^‡^	142 ± 15	153 ± 20	150 ± 13	148 ± 9	153 ± 8	0.828	**0.004**
cPAT_Thigh_ (ms)	173 ± 16^†^	163 ± 18	157 ± 15	161 ± 19	148 ± 18^†^	157 ± 17	0.057	**0.006**
cPAT_Toe_ (ms)	305 ± 19	282 ± 19	305 ± 14	304 ± 19	300 ± 24	294 ± 26	0.192	0.105
_bf_PWV (m/s)	8.5 ± 0.5	8.7 ± 0.8	9.1 ± 1.0	9.6 ± 1.5	9.7 ± 1.7	9.4 ± 1.5	**0.013**	0.219
AOC (ml/mmHg)	3.1 ± 0.5	3.1 ± 0.8	2.6 ± 0.5	3.1 ± 0.5	2.3 ± 0.4	2.9 ± 0.3	0.448	0.163
AOD (10^–3^ mm Hg^–1^)	5.3 ± 1.6	4.6 ± 2.6	5.4 ± 2.5	5.1 ± 2.2	3.6 ± 1.3	3.1 ± 1.0	0.053	0.937
AOA_min_ (mm^2^)	417 ± 46	441 ± 69	497 ± 62	497 ± 65	547 ± 105	595 ± 113	**0.027**	0.139

*Results are sorted by three different age groups (AgeGr): AgeGr. 1, 24–27 years; AgeGr. 2, 29–36 years and AgeGr. 3, 37–55 years. Aortic SBP and aortic DBP, aortic systolic and diastolic blood pressure; SBP and DBP, brachial systolic and diastolic blood pressure; PAT, pulse wave arrival time; ICT, isovolumetric contraction time; cPAT, PAT corrected for ICT; _bf_PWV, brachial-femoral pulse wave velocity; AOC, aortic compliance; AOD, aortic distensibility; AOA_min_, minimum aortic area. Results are represented as mean values ± standard deviation. Statistical significance (p < 0.05) for main effect (AgeGr.) and interaction (AgeGr. vs. bed rest) is indicated by p-values in bold. Significant difference between PRE and HDT is indicated by ^†^(p < 0.05) and ^‡^(p < 0.001).*

**TABLE 4:** All parameters recorded before (PRE) and at the end of head down tilt bed rest (HDT).

Parameter	Men (*n* = 16)		Women (*n* = 8)		*p-*values	
	PRE	HDT	PRE	HDT	Main effect	Interaction
Aortic SBP (mmHg)	111 ± 12	111 ± 7	102 ± 8	103 ± 5	**0.017**	0.439
Aortic DBP (mmHg)	70 ± 8	81 ± 8	75 ± 5	80 ± 4	0.394	0.109
SBP (mmHg)	129 ± 10	128 ± 7	117 ± 8	116 ± 6	**0.001**	0.389
DBP (mmHg)	68 ± 8	79 ± 8	73 ± 5	78 ± 4	0.278	0.157
Pulse pressure (mmHg)	61 ± 11	50 ± 8	44 ± 4	38 ± 3	**<0.001**	0.071
Heart rate (bpm)	58 ± 5	66 ± 11	70 ± 9	74 ± 12	**0.022**	0.614
Stroke volume (ml)	107 ± 17	89 ± 13	78 ± 12	71 ± 11	**0.001**	0.289
Cardiac output (l/min)	6.1 ± 1.0	5.9 ± 1.1	5.4 ± 0.5	5.2 ± 1.0	0.243	0.726
PAT_Arm_ (ms)	155 ± 17	167 ± 19	15 ± 19	155 ± 10	0.274	0.090
PAT_Finger_ (ms)	209 ± 20	208 ± 18	199 ± 11	198 ± 15	0.257	0.981
PAT_Thigh_ (ms)	215 ± 20	223 ± 19	199 ± 12	205 ± 17	0.050	0.732
PAT_Toe_ (ms)	353 ± 23	352 ± 25	350 ± 22	346 ± 24	0.181	0.910
ICT (ms)	48 ± 7	58 ± 11	52 ± 7	54 ± 12	0.958	0.328
cPAT_Arm_ (ms)	108 ± 16	109 ± 17	98 ± 7	101 ± 7	0.177	0.588
cPAT_Finger_ (ms)	161 ± 18	150 ± 14	147 ± 11	144 ± 9	0.131	0.438
cPAT_Thigh_ (ms)	167 ± 19	164 ± 17	147 ± 12	152 ± 16	**0.024**	0.567
cPAT_Toe_ (ms)	306 ± 18	294 ± 24	298 ± 20	290 ± 20	0.115	0.405
_bf_PWV (m/s)	8.6 ± 0.6	9.0 ± 1.2	9.9 ± 1.6	9.7 ± 1.4	**0.016**	0.161
AOC (ml/mmHg)	2.7 ± 0.6	3.1 ± 0.6	2.9 ± 0.4	3.0 ± 0.4	0.588	0.304
AOD (10^–3^ mm Hg^–1^)	4.8 ± 2.0	4.4 ± 2.3	4.8 ± 2.1	4.1 ± 2.1	0.705	0.740
AOA_min_ (mm^2^)	486 ± 97	512 ± 112	471 ± 73	490 ± 82	0.384	0.879

*Aortic SBP and aortic DBP, aortic systolic and diastolic blood pressure; SBP and DBP, brachial systolic and diastolic blood pressure; PAT, pulse wave arrival time; ICT, isovolumetric contraction time; cPAT, PAT corrected for ICT; _bf_PWV, brachial-femoral pulse wave velocity; AOC, aortic compliance; AOD, aortic distensibility; AOA_min_, minimum aortic area. Results are sorted by sex and represented as mean values ± standard deviation. Statistical significance (p < 0.05) for main effect (sex) and interaction (sex vs. bed rest) is indicated by bold p-values.*

## Discussion

The important finding of our study is that 60 days strict HDTBR did not produce clinically relevant changes in _bf_PWV or cPAT in healthy persons. In fact, _bf_PWV and cPAT at different vascular beds were almost identical before and after HDTBR. However, we observed an unexpected albeit modest transient increase in aortic area following strict HDTBR, which has not been previously described.

Strict HDTBR models some aspects of space travel, particularly cardiovascular deconditioning and cephalad fluid shifts. Both responses could affect vascular aging biomarkers including PAT, PWV, or AOD. The increase in heart rate with reductions in pulse pressure and stroke volume in our study is consistent with cardiovascular deconditioning. Similar stroke volume reductions indicating cardiac deconditioning and atrophy during HDTBR have been previously described (Levine et al., 1997; Iwasaki et al., 2000; Perhonen et al., 2001; Stenger et al., 2012; Palombo et al., 2015). HDTBR also reduces cardiopulmonary fitness (Saltin et al., 1968; Wagner, 2015) and orthostatic tolerance (Guinet et al., 2009). Strict HDTBR results in significant cephalad fluid shifts indicated by retinal papillary edema (Laurie et al., 2019) and mastoid effusions (Lecheler et al., 2021). Similar changes have been reported in astronauts returning from longer duration space missions (Inglesby et al., 2020).

An increase in heart rate is commonly associated with decreased pre-ejection period, the interval between electrocardiogram Q-wave and aortic valve opening (Weissler et al., 1968). If so, uncorrected PAT could overestimate vascular stiffness. In contrast, we observed concomitant increases in HR and ICT following strict HDTBR. The phenomenon has been previously observed with HDTBR (Hodges et al., 2010) and may result from an increased cardiac afterload (Hassan and Turner, 1983), as indicated by the observed increase in aortic DBP. Thus, uncorrected PAT_Arm_ and PAT_Thigh_ underestimated aortic stiffness in our study whereas corresponding cPAT remained unchanged. Furthermore, _bf_PWV, which is an ICT independent aortic stiffness parameter, also remained unchanged. However, most PWV measurements have the potential disadvantage of being blind to vascular stiffness changes in the aortic arch (Mitchell, 2009). Yet, AOD of the ascending aorta, which is an accepted vascular stiffness marker (Redheuil et al., 2010; Dogui et al., 2011; Voges et al., 2012), was also unchanged following HDTBR. We observed a trend for aortic area to increase with HDTBR likely indicating increased vascular filling. The observation that aortic area had almost returned to baseline after fourth day recovery is consistent with changes in vascular filling rather than remodeling. Increased vascular filling tends to increase vascular stiffness measurements (Guala et al., 2019). It is, therefore, unlikely that PWV changes were masked by altered vascular loading.

The finding that PWV does not change despite 60 days bed rest deconditioning is somewhat counterintuitive because physical inactivity is associated with increased arterial stiffness (Thijssen et al., 2011), whereas mild physical exercise appears to ameliorate aortic PWV (Havlik et al., 2005). In one previous HDTBR study, which has only been published as abstract, carotid-femoral PWV increased in every participant (Fayol et al., 2019). In another study, carotid-femoral PWV was 6.9 m/s before and 6.9 m/s after 35 days HDTBR (Palombo et al., 2015). Overall, HDTBR for up to 60 days may not elicit a consistent, clinically relevant change in PWV. Among others, changes in the collagen-to-elastin ratio, collagen crosslinks (Schellinger et al., 2019), and changes in the expression of endothelial and inducible nitric oxide synthases (Cau et al., 2012) have been implicated in vascular aging. Possibly, HDTBR alone is not sufficient to drive vascular (aortic) aging in the absence of additional risk factors. Age, which affects metabolic and muscular adaptation to bed rest (Pišot et al., 2016), is a prime suspect. In contrast to our expectation, an exploratory analysis did not provide evidence that older age exacerbates aortic stiffening during HDTBR. In fact, we observed the opposite response. Another potential explanation is that aortic remodeling sufficient to cause consistently measurable PWV changes in a smaller scale study requires more than 60 days HDTBR.

An important limitation of our study is the relatively small number of participants in each intervention group limiting the statistical power to detect potential protective actions of artificial gravity. At this point we would like to point out that we had no influence onto the overall study design. Since vascular stiffness biomarkers did not deteriorate with HDTBR, artificial gravity cannot improve the outcome. Another potential limitation is that, while covering a relatively large age range, the study is too small for a detailed analysis relating HDTBR responses to age. The same is true for a sex-specific analysis. It is reassuring that we did not observe an obvious qualitative difference in the HDTBR response between women and men. Another potential limitation of the study is that we did not assess the intima media thickness of different arteries. We also did not measure pulse wave arrival time to carotid artery or its therefrom derived carotid-femoral PWV, which is considered to be the non-invasive gold standard.

Despite these limitations we suggest that 60 days HDTBR, while producing cardiovascular deconditioning and cephalad fluid shifts, do not elicit clinically relevant changes in vascular stiffness biomarkers. Furthermore, the cardiovascular adaptation to HDTBR was not affected by daily artificial gravity training. Other analyses from AGBRESA also report no relevant influences of the artificial gravity intervention on physiological outcomes (Attias et al., 2020; Hoffmann et al., 2020; Kramer et al., 2020; Ganse et al., 2021; Lecheler et al., 2021). Combining artificial gravity with exercise holds promise for future bed rest studies (Diaz-Artiles et al., 2018).

Clearly, our findings cannot be simply extrapolated to real space conditions. In fact, current missions in low Earth orbit and future missions to Mars are substantially longer. Moreover, astronauts are exposed to additional risks that could exacerbate vascular aging, particularly radiation (Hughson et al., 2018). Our findings may also help interpreting previous vascular function data obtained in space and guide technology development for future missions. For example, previous studies in astronauts reported reduced PAT without measuring ICT (Baevsky et al., 2007; Hughson et al., 2016). Cardiac deconditioning in space (Hughson et al., 2018) could conceivably affect ICT. In fact, prolonged pre-ejection periods were reported in three Skylab astronauts after 59 days in space (Bergman et al., 1976). Combined with our findings from HDTBR, we conclude that only ICT corrected PAT values should be used as vascular biomarkers.

## Data Availability Statement

The raw data supporting the conclusions of this article will be made available by the authors, without undue reservation.

## Ethics Statement

The studies involving human participants were reviewed and approved by the Ethics Commissions of the Medical Association North Rhine (number 2018143) and NASA (Johnson Space Center, Houston, United States). The patients/participants provided their written informed consent to participate in this study.

## Author Contributions

SM, JT, MB, and BH designed the experiment. MB provided technical support. SM, FH, and JR performed data acquisition. SM, FH, and SO were in charge of data analysis. BJ did statistical analysis. SM was primarily in charge of drafting the manuscript. EM, JT, and JJ primarily revised the manuscript. All authors contributed and accepted the final version.

## Conflict of Interest

The authors declare that the research was conducted in the absence of any commercial or financial relationships that could be construed as a potential conflict of interest.
